# Domain adaptation spatial feature perception neural network for cross-subject EEG emotion recognition

**DOI:** 10.3389/fnhum.2024.1471634

**Published:** 2024-12-17

**Authors:** Wei Lu, Xiaobo Zhang, Lingnan Xia, Hua Ma, Tien-Ping Tan

**Affiliations:** ^1^Henan High-speed Railway Operation and Maintenance Engineering Research Center, Zhengzhou Railway Vocational and Technical College, Zhengzhou, China; ^2^School of Computer Sciences, Universiti Sains Malaysia, Penang, Malaysia; ^3^Jiangxi Vocational College of Finance and Economics, Jiujiang, China

**Keywords:** affective computing, electroencephalography, emotion recognition, convolutional neural network, graph attention network, domain adaptation

## Abstract

Emotion recognition is a critical research topic within affective computing, with potential applications across various domains. Currently, EEG-based emotion recognition, utilizing deep learning frameworks, has been effectively applied and achieved commendable performance. However, existing deep learning-based models face challenges in capturing both the spatial activity features and spatial topology features of EEG signals simultaneously. To address this challenge, a **d**omain-adaptation **s**patial-feature **p**erception-network has been proposed for cross-subject EEG emotion recognition tasks, named DSP-EmotionNet. Firstly, a **s**patial **a**ctivity **t**opological **f**eature **e**xtractor **m**odule has been designed to capture spatial activity features and spatial topology features of EEG signals, named SATFEM. Then, using SATFEM as the feature extractor, DSP-EmotionNet has been designed, significantly improving the accuracy of the model in cross-subject EEG emotion recognition tasks. The proposed model surpasses state-of-the-art methods in cross-subject EEG emotion recognition tasks, achieving an average recognition accuracy of 82.5% on the SEED dataset and 65.9% on the SEED-IV dataset.

## Introduction

Emotion recognition (Jia et al., 2021; Tan et al., 2020; Cimtay et al., 2020; Doma and Pirouz, 2020) has become an important task in affective computing. It has potential applications in areas like affective brain-computer interfaces, diagnosing affective disorders, detecting emotions in patients with consciousness disorders, emotion detection of drivers, mental workload estimation, and cognitive neuroscience. Emotion is a mental and physiological state that arises from various sensory and cognitive inputs, significantly influencing human behavior in daily life (Jia et al., 2021). Emotion is a response to both internal and external stimuli. Physiological signals, such as Electrocardiography (ECG), Electromyography (EMG), and Electroencephalography (EEG), correspond to the physiological responses caused by emotions. They are more reliable indicators of emotional expression than non-physiological signals, such as speech, posture, and facial expression, which can be masked by humans (Tan et al., 2020; Cimtay et al., 2020). Among these physiological signals, EEG signals have a high temporal resolution and a wealth of information, which can reveal subtle changes in emotions, making them more suitable for emotion recognition than other physiological signals (Atkinson and Campos, 2016). EEG-based emotion recognition methods are more accurate and objective, as some studies have verified the relationship between EEG signals and emotions (Xing et al., 2019).

In recent years, EEG signals have gained widespread application in emotion recognition due to their ability to accurately reflect the genuine emotions of subjects (Jia et al., 2020; Zhou et al., 2023). Early approaches to EEG-based emotion recognition have relied on processes such as signal denoising, feature design, and classifier learning. For example, Wang et al. have introduced the Support Vector Machine (SVM) classifier (Wang et al., 2011), while Bahari et al. have proposed the K-Nearest Neighbors (KNN) classifier (Bahari and Janghorbani, 2013), both achieving effective emotion classification. However, traditional machine learning techniques have been constrained by intricate feature engineering and selection processes. To overcome these limitations, researchers have introduced deep learning techniques. The continuous refinement of deep learning algorithms has led to significant achievements in EEG-based emotion recognition. For example, Kwon et al. have utilized CNN to extract features from EEG signals, while Li et al. have obtained deep representations of all EEG electrode signals using Recurrent Neural Networks (RNN; Kwon et al., 2018; Li et al., 2020). Additionally, some researchers have adopted hybrid models combining Convolutional Neural Networks (CNN) and RNN. For instance, Ramzan et al. have proposed a parallel CNN and LSTM-RNN deep learning model for emotion recognition and classification (Ramzan and Dawn, 2023). Although traditional neural network models such as CNN and RNN have achieved high accuracy in EEG emotion recognition tasks, they typically process data in the form of grid data. However, grid data cannot effectively represent connections between different brain regions, thus hindering models from directly capturing the spatial topological features of EEG signals. To better capture connections between brain regions and achieve improved performance in emotion recognition tasks, researchers have begun exploring the use of graph data to represent interactions between brain regions and employing Graph Neural Networks (GNNs) to process this data. For instance, Asadzadeh et al. have proposed an emotion recognition method based on EEG source signals using a Graph Neural Network approach (Asadzadeh et al., 2023). However, models based on GNNs face challenges in accurately detecting local features and capturing the spatial activity features of EEG signals.

However, when applying deep learning models to interdisciplinary tasks such as EEG-based emotion recognition, significant challenges arise due to the limited number of subjects in EEG emotion datasets, coupled with individual differences among subjects. This often results in a notable decrease in the performance of deep learning models in cross-subject EEG emotion recognition tasks. To address the issue of poor performance of subjects in EEG emotion recognition, many researchers have begun exploring the application of transfer learning techniques. In cross-subject EEG emotion recognition tasks, transfer learning primarily addresses the issue of domain gaps caused by individual differences. Transfer learning mainly includes fine-tuning and domain adaptation. Fine-tuning, as an effective knowledge transfer method, has gained widespread adoption. Zhang et al. introduced the Self-Training Maximum Classifier Difference (SMCD) model, utilizing fine-tuning to apply a model trained on the source domain to the target domain (Zhang et al., 2023). However, collecting a large amount of labeled data from the target domain requires considerable time, manpower, and financial resources. Especially in tasks like EEG emotion recognition, acquiring large-scale EEG datasets and labeling them is a complex and expensive task. In some cases, labeled data from the target domain may be extremely scarce, or even insufficient for fine-tuning, which limits the performance and generalization ability of the model on the target task. Researchers have begun exploring the application of domain adaptation in cross-disciplinary EEG emotion recognition. Li et al. proposed a Domain Adaptation method that enhances adaptability by minimizing source domain error and aligning latent representations (Li et al., 2019). However, the majority of existing domain adaptation methods only focus on extracting shallow-level features, without effectively aligning deep-level features of different types. This greatly limits the ability of the model for cross-domain transfer learning.

The primary contributions of this paper can be outlined as follows:

- To accurately capture the activity states of different brain regions and their inter-regional connectivity, we have designed a dual-branch **S**patial **A**ctivity **T**opological **F**eature **E**xtractor **M**odule, named SATFEM. This module has been able to simultaneously extract spatial activity features and spatial topological features from EEG signals, significantly enhancing the recognition performance of the model.
- To minimize the disparity between the source and target domains, we have devised a **D**omain-adaptation **S**patial-feature **P**erception-network for cross-subject EEG emotion recognition, resulting in the proposal of the DSP-EmotionNet model. This model is tailored to enhance the generalization of the model on the target domain, thereby elevating the accuracy of cross-subject EEG emotion recognition tasks.
- The proposed DSP-EmotionNet model achieves accuracy rates of 82.5% and 65.9% on the SEED and SEED-IV datasets, respectively, for cross-subject EEG emotion recognition tasks. These rates surpass those of state-of-the-art models. Additionally, a series of ablation experiments have been conducted to investigate the contributions of key components within DSP-EmotionNet to the recognition performance of cross-subject EEG emotion recognition tasks.

## 1 Related work

Traditional EEG feature extractors, such as CNNs and RNNs, have limitations in capturing the connections between brain regions, which constrains their ability to extract spatial topological features. Although GNN models have made improvements in this area, they still face challenges in detecting subtle local variations. Domain adaptation techniques have shown success in cross-subject EEG emotion recognition tasks, but most existing domain adaptation-based methods focus predominantly on aligning shallow features, failing to effectively utilize deeper and more diverse feature types.

### 1.1 EEG spatial activity feature extractor

In recent years, the application of EEG signals in the field of emotion recognition has significantly increased. This is mainly attributed to the accurate and authentic reflection of the true emotional states of individuals by EEG signals. With the development of deep learning, two popular deep learning models, CNN and RNN, have been widely applied in EEG emotion recognition. For instance, Kwon et al. have utilized CNN for feature extraction from EEG signals. In their model, the EEG signal undergoes preprocessing via wavelet transform before convolution, considering both the time and frequency aspects of the EEG signal (Kwon et al., 2018). Li et al. have employed four directed RNNs based on two spatial directions to traverse the electrode signals of two different brain regions, obtaining a deep representation of all EEG electrode signals while preserving their inherent spatial dependencies (Li et al., 2020). Moreover, some researchers have adopted hybrid models combining CNN and RNN. For example, Chakravarthi et al. have proposed a classification method that combines CNN and LSTM, aiming to recognize and classify different emotional states by analyzing EEG data (Chakravarthi et al., 2022). Ramzan et al. have proposed a parallel CNN and Long Short-Term Memory Recurrent Neural Network (LSTM-RNN) deep learning model, which primarily utilizes CNN for extracting spatial features of EEG signals and LSTM-RNN for extracting temporal features of EEG signals, thus achieving emotion recognition and classification (Ramzan and Dawn, 2023). However, EEG spatial activity feature extractors such as CNNs and RNNs typically process data in a grid format. While grid data can effectively reflect the spatial activity states of EEG signals, it fails to adequately represent the connections between different brain regions. This limitation hinders the model's ability to directly capture the spatial topological features of EEG signals.

### 1.2 EEG spatial topological feature extractor

Despite the high accuracy achieved by traditional neural network models such as CNN and RNN in EEG emotion recognition tasks, the data they handle is typically in the form of grid data. EEG data are usually captured from multiple electrodes on the scalp, with each electrode signal representing the activity of the corresponding brain region. However, grid data cannot effectively represent the connectivity between brain regions, thereby preventing the model from directly capturing the connections between different brain regions. Therefore, in order to better capture the connectivity between brain regions and achieve better performance in emotion recognition tasks, researchers have begun to explore the use of graph data to represent the connections between brain regions and leverage GNNs to process such graph data. For instance, Asadzadeh et al. have proposed an emotion recognition method based on EEG source signals using a Graph Neural Network node (ESB-G3N). This method treats EEG source signals as node signals in graph data, the relationships between EEG source signals as the adjacency matrix of the graph data and employs GNN for EEG emotion recognition (Asadzadeh et al., 2023). However, GNN-based models have certain advantages as EEG spatial topological feature extractors in processing the spatial topological features of EEG signals, they face challenges in accurately detecting local features and subtle variations in brain activity.

### 1.3 Transfer learning for emotion recognition

Due to the potential applications of deep learning models in various fields, there is great interest in utilizing these models for EEG-based emotion recognition. However, when applying deep learning models to cross-subject EEG emotion recognition tasks, there is a significant challenge due to the limited number of subjects in EEG emotion datasets, coupled with individual differences between subjects. This often results in a significant drop in the performance of deep learning models in interdisciplinary EEG emotion recognition tasks. To address the issue of decreased performance of subjects in EEG emotion recognition, many researchers have begun to explore the application of transfer learning techniques. In interdisciplinary EEG emotion recognition tasks, transfer learning primarily addresses the problem of data domain gaps caused by individual differences. EEG signals from different subjects in the same emotional state may exhibit significant variations due to individual differences. In such cases, the target domain has represented the feature space of EEG data obtained from a certain number of subjects. In contrast, the source domain has included data collected from one or more different individuals. Li et al. have incorporated fine-tuning into emotion recognition networks and examined the extent to which the models can be shared among subjects (Li et al., 2018). Wang et al. have proposed a method that utilizes fine-tuning to address the challenge of emotional differences across different datasets in deep model transfer learning, to construct a robust emotion recognition model (Wang et al., 2020). These methods overcome subject differences by training on the source domain and fine-tuning on the target domain. Although existing transfer learning methods for EEG emotion recognition can achieve improved results, almost all existing work requires the use of a certain amount of labeled data from the target domain for fine-tuning training. However, collecting a large amount of labeled data from the target domain requires a considerable amount of time, manpower, and financial resources. Especially in tasks such as EEG emotion recognition, obtaining large-scale EEG datasets and labeling them is a complex and expensive task. In some cases, the labeled data from the target domain may be extremely scarce or even insufficient for fine-tuning, which may limit the performance and generalization ability of the model on the target task. Therefore, some researchers have begun exploring the application of domain adaptation for cross-subject eeg emotion recognition. For example, Jin et al. have proposed the utilization of the Domain Adaptation Network (DAN) for knowledge transfer in EEG-based emotion recognition to address the fundamental problem of mitigating differences between the source subject and target subject in order to eliminate subject variability (Jin et al., 2017). Li et al. have proposed a domain adaptation method for EEG emotion recognition, which is optimized by minimizing the classification error on the source domain while simultaneously aligning the latent representations of the source and target domains to make them more similar (Li et al., 2019). Wang et al. have proposed an efficient few-label domain adaptation method based on the multi-subject learning model for cross-subject emotion classification tasks with limited EEG data (Wang et al., 2021). However, most existing domain adaptation-based methods for cross-subject EEG emotion recognition focus primarily on aligning shallow features, without effectively aligning and fully utilizing deeper, more diverse types of features.

## 2 Methodology

### 2.1 Overview

**Figure 1:**
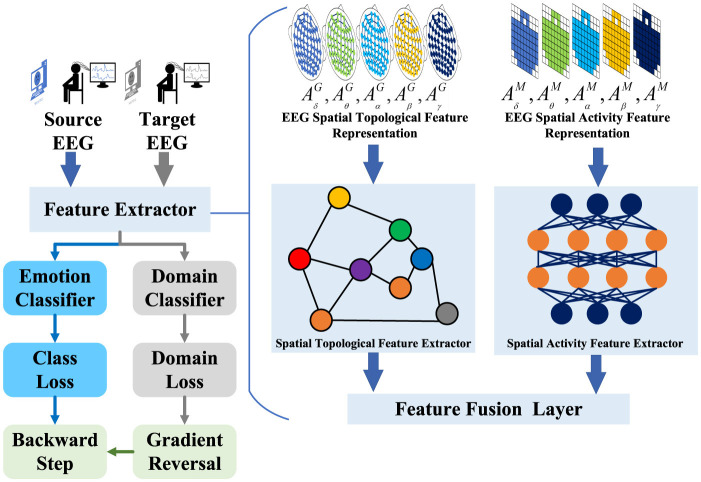
The overall architecture of DSP-EmotionNet for EEG emotion recognition is as follows. Initially, two distinct feature maps of the brain are constructed: one representing EEG spatial activity features and the other representing EEG spatial topological features. Subsequently, the spatial activity feature extractor is employed to detect subtle changes in brain activity, and the spatial topological feature extractor is used to capture the connectivity between different brain regions. Finally, a domain adaptation spatial feature perception network is proposed for cross-subject EEG emotion recognition tasks, aimed at enhancing the generalization capability of the model.

The overall architecture of the proposed model is illustrated in Figure 1. We summarize three key ideas of the proposed DSP-EmotionNet model as follows: (1) Constructing EEG spatial activity features and EEG spatial topological features. (2) Integrating spatial activity feature extractor and spatial topological feature extractor to capture the connections between different brain regions and the subtle changes in brain activity, this module is named the SATFEM module. The SATFEM module enhances the generalization ability of the model in cross-subject EEG emotion recognition by extracting both spatial activation and spatial topological features, resulting in a more robust feature representation. Compared to traditional methods that focus on a single type of feature, this combined approach better captures the complexity of EEG data. (3) Utilizing the SATFEM module as a feature extractor, a domain adaptation spatial feature perception network was proposed for cross-subject EEG emotion recognition tasks, improving the generalization ability of the model. This method not only applies domain adaptation techniques but also employs a dual-branch feature extractor to ensure effective domain feature alignment between different subjects. This enables domain adaptation to go beyond merely aligning shallow features, allowing for the effective alignment of deeper and more diverse feature types.

### 2.2 EEG feature representations

In this section, we introduce two distinct EEG feature representations: EEG spatial activity feature representation and EEG spatial topological feature representation. These different feature representations reflect various spatial relationships within the brain. Specifically, We employ EEG spatial activity feature representation to illustrate spatial activation state distribution maps of the brain, which can reflect the activation states of different brain regions in space. We use EEG spatial topological feature representation to depict spatial topological functional connectivity maps of the brain, which can reflect the connectivity between different brain regions in space. These two EEG feature representations complement each other and effectively demonstrate the spatial relationships of EEG signals.

#### 2.2.1 EEG spatial activity feature representation

**Figure 2:**
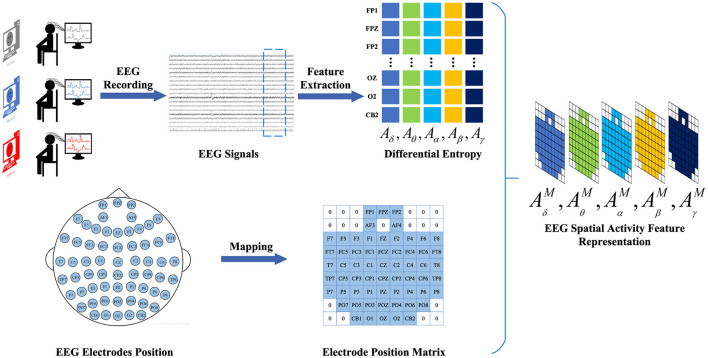
The construction process of EEG spatial activity feature representation. We adopt a time-frequency feature extraction method to extract 4-s EEG signal DE features from EEG signal samples. Subsequently, based on the electrode positions of the brain, the selected data are mapped onto the brain electrode position matrix. Finally, the electrode position matrices corresponding to different frequencies are superimposed to generate a spatial activity feature representation of the EEG signal.

To construct the EEG spatial activity feature representation, we employ the temporal-frequency feature extraction method to derive the Differential Entropy (DE) of five frequency bands {δ, θ, α, β, γ} from all EEG channels across EEG signal samples within 4-s segments. We denote $A_{B}=\left(A_{\delta },A_{\theta },A_{\alpha },A_{\beta },A_{\gamma }\right)\in {\mathbb{R}}^{N_{e}\times B}$ as a frequency feature matrix comprising frequency bands extracted from the DE feature, where *B* ∈ {δ, θ, α, β, γ} represents the frequency band and *N*_*e*_ ∈ {*FP*1, *FPZ*, ..., *CB*2} denotes the electrode. Subsequently, the selected data are mapped onto a frequency domain brain electrode location matrix $A_{b}^{M}\in {\mathbb{R}}^{H\times W},\left(b\in \left\{1,2,...,B\right\}\right)$, based on the electrode positions of the brain. Finally, the frequency-domain brain electrode position matrices corresponding to different frequencies are overlaid to generate the spatial-frequency feature representation of EEG signals. Thus, the construction of the EEG feature representation $A^{M}=\left(A_{\delta }^{M},A_{\theta }^{M},A_{\alpha }^{M},A_{\beta }^{M},A_{\gamma }^{M}\right)\in {\mathbb{R}}^{H\times W\times B}$ is completed. The construction process of EEG spatial activity feature representation is illustrated in Figure 2.

#### 2.2.2 EEG spatial topological ferture representation

**Figure 3:**
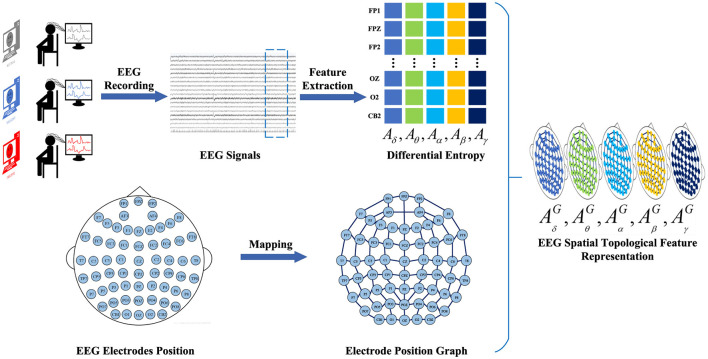
The construction process of EEG spatial topological feature representation. We employ a time-frequency feature extraction method to extract 4-s EEG signal DE features from EEG signal samples. Subsequently, the brain electrode position network is defined as a graph representation. Finally, the graph representations of electrode positions corresponding to different frequencies are superimposed to generate a spatial topological feature representation of the EEG signal.

To construct the EEG spatial topological feature representation, we employ the temporal-frequency feature extraction method to derive the DE of five frequency bands {δ, θ, α, β, γ} from all EEG channels across EEG signal samples within 4-s segments. We denote $A_{B}=\left(A_{\delta },A_{\theta },A_{\alpha },A_{\beta },A_{\gamma }\right)\in {\mathbb{R}}^{N_{e}\times B}$ as a frequency feature matrix comprising frequency bands extracted from the DE feature, where *B* ∈ {δ, θ, α, β, γ} represents the frequency band and *N*_*e*_ ∈ {*FP*1, *FPZ*, ..., *CB*2} denotes the electrode. Subsequently, the frequency domain brain electrode network is defined as a graph *G* = (*V, E, A*), where *V* represents the set of vertices, with each vertex representing an electrode in the brain; *E* denotes the set of edges, indicating the connections between vertices; and *A* denotes the adjacency matrix of the brain electrode network *G*. Finally, the frequency-domain brain electrode position graph corresponding to different frequencies is overlaid to generate the spatial-frequency feature representation of EEG signals. Thus, the construction of the EEG feature representation $A^{G}=\left(A_{\delta }^{G},A_{\theta }^{G},A_{\alpha }^{G},A_{\beta }^{G},A_{\gamma }^{G}\right)$ is completed. The construction process of EEG spatial topological feature representation is illustrated in Figure 3.

### 2.3 Spatial feature perception extractor

Using EEG spatial activity features and EEG spatial topological features as inputs, a dual-branch spatial-activity-topological feature extractor module named SATFEM is designed. SATFEM can simultaneously extract spatial activity features and spatial topological features. The features extracted from the dual branches are fused at the feature fusion layer. Algorithm 1 shows the pseudocode for SATFEM. The SATFEM feature extractor consists of three main components: the spatial-topological feature extractor, the spatial activity feature extractor, and the feature fusion layer.

**Algorithm 1:**
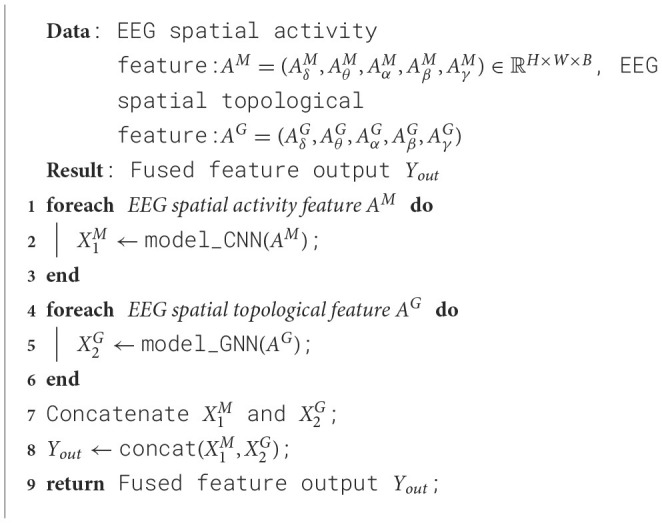
SATFEM.

#### 2.3.1 Spatial topological feature extractor

The Graph Attention Network (GAT) is proposed to address issues in deep GNN models, such as inefficient information propagation and unclear relationships between nodes (Velickovic et al., 2017). GAT utilizes attention mechanisms to dynamically allocate weights between nodes, thereby enhancing the influence of important nodes and improving the efficiency of information propagation and clarity of relationships between nodes. Therefore, it is suitable for extracting EEG spatial topological feature representation as a feature extractor. This helps capture relationships between different functional areas in EEG feature representation, facilitating more accurate identification of different EEG signals. The input of GAT is the EEG spatial topological feature representation $A^{G}=\left(A_{\delta }^{G},A_{\theta }^{G},A_{\alpha }^{G},A_{\beta }^{G},A_{\gamma }^{G}\right)$.

In graph, let any node *v*_*i*_ in the *l* − *th* layer correspond to the feature vector *h*_*i*_, where $h_{i}\in {\mathbb{R}}^{d^{\left(l\right)}}$, and *d*^(*l*)^ represents the feature dimension of the node. After an aggregation operation centered around the attention mechanism, the output is the new feature vector ${h_{i}}^{\prime }$, where ${h_{i}}^{\prime }\in {\mathbb{R}}^{d^{\left(l+1\right)}}$, and *d*^(*l*+1)^ represents the length of the output feature vector. This aggregation operation is called the Graph Attention Layer(GAL).

Assuming the central node is *v*_*i*_, let the weight coefficient from neighboring node *v*_*j*_ to *v*_*i*_ be denoted as Equation 1.


(1) $$\begin{array}{l} e_{ij}=\alpha \left(Wh_{i},Wh_{j}\right), \end{array}$$

The weight parameter *W* ∈ ℝ^*d*^(*l*+1)^×*d*^(*l*)^^ is used for the feature transformation of nodes in this layer. α(·) is the function used to compute the correlation between two nodes. The fully connected layer for a single layer is described as Equation 2.


(2) $$\begin{array}{l} e_{ij}=\text{LeakyReLU}\left(\alpha^{\text{T}}\left[Wh_{i}∥Wh_{j}\right]\right), \end{array}$$

where the weight parameter α ∈ ℝ^2*d*^(*l*+1)^^, and the activation function is designed as the LeakyReLU function. To better distribute weights, it is necessary to uniformly normalize the relevance computed with all leaders, specifically through softmax normalization as shown in Equation 3.


(3) $$\begin{array}{l} \alpha_{ij}=\text{softma}{\text{x}}_{j}\left(e_{ij}\right)=\frac{\exp\left(e_{ij}\right)}{\sum_{v_{k}\in Ñ\left(v_{i}\right)}\exp\left(e_{ik}\right)}, \end{array}$$

The weight coefficient α is calculated such that Equation 3 ensures that the sum of the weight coefficients for all neighbors is equal to 1. The complete formula for calculating the weight coefficients is described in Equation 4.


(4) $$\begin{array}{l} \alpha_{ij}=\frac{\exp\left(\text{LeakyReLU}\left(\alpha^{\text{T}}\left[Wh_{i}∥Wh_{j}\right]\right)\right)}{\sum_{v_{k}\in Ñ\left(v_{i}\right)}\exp\left(\text{LeakyReLU}\left(\alpha^{\text{T}}\left[Wh_{i}∥Wh_{k}\right]\right)\right)}, \end{array}$$

Following the calculation of the weight coefficients as described above, according to the weighted sum with attention mechanism, the new feature vector of node *v*_*i*_ is obtained as shown in Equation 5.


(5) $$\begin{array}{l} {h_{i}}^{\prime }=\sigma \left(\underset{v_{j}\in Ñ\left(v_{i}\right)}{\sum }\alpha_{ij}Wh_{j}\right), \end{array}$$

#### 2.3.2 Spatial activity feature extractor

The Residual Network (ResNet) is proposed to address the problem of degradation in deep CNN models. ResNet utilizes residual connections to link different convolutional layers, thereby enabling the propagation of shallow feature information to the deeper layers. Therefore, it is suitable for extracting EEG spatial Activity feature representation as a feature extractor.

The input of ResNet is the EEG spatial Activity feature representation $A^{M}=\left(A_{\delta }^{M},A_{\theta }^{M},A_{\alpha }^{M},A_{\beta }^{M},A_{\gamma }^{M}\right)\in {\mathbb{R}}^{H\times W\times B}$. The EEG spatial Activity feature representation first goes through *conv*1, which consists of a 7 × 7 convolutional layer, a max pooling operation, and Batch Normalization. The *conv*1 layer is responsible for the initial processing of spatial information extraction and feature representation for EEG spatial Activity feature representation. Specifically, the input of *conv*1 is the spatial-frequency feature representation $A^{M}=\left(A_{\delta }^{M},A_{\theta }^{M},A_{\alpha }^{M},A_{\beta }^{M},A_{\gamma }^{M}\right)\in {\mathbb{R}}^{H\times W\times B}$, where the shape of the spatial Activity feature representation is *H* × *W* × *C*, with *H* representing the height, *W* representing the width, and *C* representing the number of channels. Due to the number of frequency bands being 5, *C* = 5. However, this does not meet the input requirements of the original ResNet model, as the first convolutional layer in the original ResNet model requires an input channel size of 3. If the original model is used directly to process data with 5 input channels, channel conversion or padding operations are required, which may result in the loss of important information from the original data. Therefore, we replaced the first half of the ResNet model with a new convolutional layer that has 5 input channels, 64 output channels, a kernel size of 7 × 7, a stride of 2, a padding of 4, and no bias. The equations for *conv*1 of ResNet are shown in equations Equation 6.


(6) $$\begin{array}{l} C_{1}=\text{MaxPool}\left(\text{ReLU}\left(\text{BN}\left(\text{Con}{\text{v}}_{7\times 7}\left(A^{M}\right)\right)\right)\right), \end{array}$$

where *A*^*M*^ is the input of *conv*1 in the CNN branch, *C*1 is the output of conv1 in the CNN branch. Conv_7 × 7_(·) represents the convolutional layer operation with an output channel of 64, kernel size of 7 × 7, the stride of 2, and padding of 4. BN(·) represents the batch normalization layer operation, which performs batch normalization on the output of the convolutional layer. ReLU(·) represents the ReLU activation function, which applies the ReLU activation function to the output of the batch normalization layer. MaxPool(·) represents the max pooling layer operation, which performs max pooling using a 3 × 3 pooling kernel, a stride of 2, and padding of 1.

The features output from *conv*1 are processed through *conv*2_*x*_, *conv*3_*x*_, *conv*4_*x*_, and *conv*5_*x*_, respectively. Each of *conv*2_*x*_, *conv*3_*x*_, *conv*4_*x*_, and *conv*5_*x*_ consists of 2 BasicBlocks. In BasicBlock, the input feature is added to the main branch output feature via a shortcut connection before being passed through a ReLU activation function. The equation of the main branch, as shown in Equation 7.


(7) $$\begin{array}{l} X_{main}=\text{BN}\left(\text{Con}{\text{v}}_{3\times 3}\left(\text{ReLU}\left(\text{BN}\left(\text{Con}{\text{v}}_{3\times 3}\left(X_{BasicIN}\right)\right)\right)\right)\right), \end{array}$$

where *X*_*BasicIN*_ is the input of BasicBlock, *X*_*main*_ is the output of the main branch in the BasicBlock. Conv_3×3_(·) represents the convolutional layer operation with a kernel size of 3 × 3, the stride of 1, and padding of 1. BN(·) represents the batch normalization layer operation, which performs batch normalization on the output of the convolutional layer. ReLU(·) represents the ReLU activation function, which applies the ReLU activation function to the output of the batch normalization layer.

The shortcut connection allows the gradient to flow directly through the network, bypassing the convolutional layers in the main branch, which helps to prevent the vanishing gradient problem. The equation of the shortcut connection, as shown in Equation 8.


(8) $$\begin{array}{l} X_{shortcut}==\text{BN}\left(\text{Con}{\text{v}}_{1\times 1}\left(\text{Con}{\text{v}}_{3\times 3}\left(X_{BasicIN}\right)\right)\right), \end{array}$$

where *X*_*BasicIN*_ is the input of BasicBlock, *X*_*shortcut*_ is the output of the shortcut connection in the BasicBlock. Conv_3×3_(·) represents the convolutional layer operation with a kernel size of 3 × 3, the stride of 1, and padding of 1. Conv_1 × 1_(·) represents the convolutional layer operation with a kernel size of 1 × 1. BN(·) represents the batch normalization layer operation. ReLU(·) represents the ReLU activation function.

The addition of the input feature to the main branch output feature allows the network to learn residual mappings, which can be easier to optimize during training. The equation of the addition, as shown in Equation 8.


(9) $$\begin{array}{l} X_{BasicOUT}=\text{ReLU}\left(X_{main}+X_{shortcut}\right), \end{array}$$

where *X*_*main*_ is the output of the main branch, *X*_*shortcut*_ is the output of the shortcut connection, and *X*_*BasicOUT*_ is the output of the BasicBlock. ReLU(·) represents the ReLU activation function.

#### 2.3.3 Feature fusion layer

Utilizing EEG spatial activity feature representation and EEG spatial topological feature representation, the EEG spatial activity extractor and EEG spatial topological feature extractor respectively extract local features of EEG signals and functional connectivity of brain regions from EEG signals. Subsequently, the extracted EEG spatial activity feature representation information and EEG spatial topological feature representation information are fused in the Feature Fusion layer, as outlined in Equation 10. The fused dual-branch network module is referred to as the spatial-activity-topology feature extraction network module, abbreviated as SATFEM.


(10) $$\begin{array}{l} Y_{out}=\text{Fusion}\left(X_{1}^{M}∥X_{2}^{G}\right) \end{array}$$

where ∥ represents the concatenate operation,$X_{1}^{M}$, and $X_{2}^{G}$ respectively represent the EEG spatial activity feature and EEG spatial topological feature features extracted by the EEG spatial activity extractor and EEG spatial topological feature extractor branches, *Y*_*out*_ represents the fused feature output.

### 2.4 Domain adaptation

A Domain Adversarial Neural Network (DANN) is used for implementing transfer learning. This framework was initially proposed by Ganin et al. for image classification (Ganin and Lempitsky, 2015). Building upon the original DANN model, a domain adaptive learning model for EEG emotion recognition is proposed, utilizing SATFEM as the feature extractor, named DSP-EmotionNet. The aim is to address domain differences among different subjects. Algorithm 2 shows the pseudocode for DSP-EmotionNet. The architecture of this model comprises three main components: the feature extractor, emotion classifier, and domain classifier.

**Algorithm 2:**
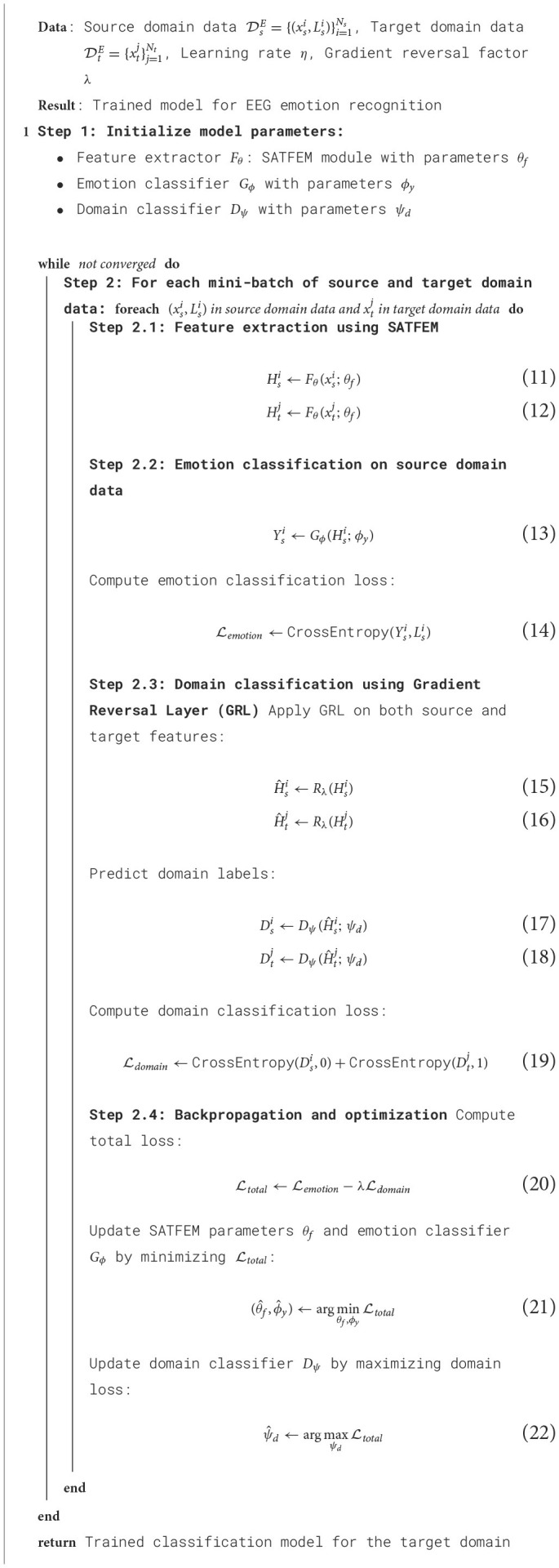
DSP-EmotionNet.

The feature extractor is used to extract shared EEG emotion representations from both the source and target domain input data. For the model, the SATFEM module is selected as the feature extractor. The formula for the feature extractor in the model can be represented as Equation 23:


(23) $$\begin{array}{l} H_{i}=F_{\theta }\left(x_{i};\theta_{f}\right) \end{array}$$

where *x*_*i*_ represents the input sample, while *H*_*i*_ represents the output feature representation obtained from the feature extractor. The feature extractor utilizes parameters θ_*f*_ to map the input sample *x*_*i*_ to a high-level feature space that contains abstract features useful for the adversarial transfer learning task of EEG-based emotion recognition. These features are then passed to the emotion classifier and domain classifier for subsequent emotion recognition and domain adaptive learning tasks.

The emotion classifier is a classifier used for emotion classification. It takes the shared features extracted by the feature extractor as input and performs emotion classification on the source domain data. In this case, a fully connected layer is chosen as the classifier for emotion classification. The formula for the emotion classifier in the model can be represented as Equation 24:


(24) $$\begin{array}{l} Y_{i}=G_{\phi }\left(H_{i};\phi_{y}\right) \end{array}$$

where *H*_*i*_ represents the output feature representation from the feature extractor, and *Y*_*i*_ represents the emotion prediction results of the model for the input sample *x*_*i*_. The emotion classifier maps the feature representation *H*_*i*_ to a predicted probability distribution over emotion labels using the parameter ϕ_*y*_.

The domain classifier is used to determine whether the input features are from the source domain or the target domain. It takes the shared features extracted by the feature extractor as input and attempts to correctly classify them as belonging to the source domain or the target domain. The objective of the domain classifier, achieved through adversarial training, is to make the extracted features indistinguishable in terms of the domain. The formula for the Domain Classifier in the model can be represented as Equation 25:


(25) $$\begin{array}{l} D_{i}=D_{\psi }\left(H_{i};\psi_{d}\right) \end{array}$$

where *H*_*i*_ represents the output feature representation from the Feature Extractor, and *D*_*i*_ represents the prediction results of the domain label for the input sample *x*_*i*_. The Domain Classifier maps the feature representation *H*_*i*_ to a predicted probability distribution over domain labels using the parameter ψ_*d*_.

The model is capable of learning universal feature representations from EEG emotion data of different subjects, thereby improving the emotion recognition performance of both the source and target domains. Through domain adaptation training, this transfer learning model aligns the feature representations of the source and target domains, further enhancing the generalization ability and adaptability of the model to the target domain. The overall training objective of the model can be expressed as Equation 26.


(26) $$\begin{array}{l} \begin{array}{c} E\left(\theta_{f},\phi_{y},\psi_{d}\right)=\underset{x_{i}\in D_{s}^{E}}{\sum }L_{emotion}\left(G_{\phi }\left(F_{\theta }\left(x_{i}\right)\right),L_{i}^{y}\right)\\ -\lambda \underset{x_{i}\in D_{s}^{E}\cup D_{t}^{E}}{\sum }L_{domain}\left(D_{\psi }\left(F_{\theta }\left(x_{i}\right)\right),L_{i}^{d}\right) \end{array} \end{array}$$

where θ_*f*_, ϕ_*y*_, and ψ_*d*_ represent the parameters of the feature extractor *F*_θ_, the emotion classifier *G*_ϕ_, and the domain classifier *D*_ψ_, respectively. $L_{emotion}$ denotes the emotion classification loss, while $L_{domain}$ represents the domain classification loss. The emotion samples are denoted by *x*_*i*_, and $L_{i}^{y}$ represents their corresponding true emotion labels. Additionally, $L_{i}^{d}$ represents their corresponding domain labels, where $L_{i}^{d}=0$ indicates that the sample *x*_*i*_ comes from the source domain, and $L_{i}^{d}=1$ indicates that the sample *x*_*i*_ comes from the target domain.

The model first optimizes the parameters θ_*f*_ and ϕ_*y*_ of the feature extractor *F*_θ_ and emotion classifier *G*_ϕ_ by minimizing the classification loss and the feature extractor loss. This is achieved through the following formula, as shown in Equation 27:


(27) $$\begin{array}{l} \left({\hat{\theta }}_{f},{\hat{\phi }}_{y}\right)=\underset{\theta_{f},\phi_{y}}{\arg\min}E\left(\theta_{f},\phi_{y},\psi_{d}\right) \end{array}$$

Then, the model optimizes the parameters ψ*d* of the domain classifier *Dψ* by maximizing its loss. This is achieved through the following formula, as shown in Equation 28:


(28) $$\begin{array}{l} \left({\hat{\psi }}_{d}\right)=\underset{\psi_{d}}{\arg\max}E\left(\theta_{f},\phi_{y},\psi_{d}\right) \end{array}$$

The two steps mentioned above are alternated until the network converges. During the domain adaptive learning process, a gradient reversal layer is employed to induce the feature extractor to learn adversarial feature representations, as shown in Equation 29:


(29) $$\begin{array}{l} R_{\lambda }\left(x\right)=x \end{array}$$

During backpropagation, the gradient reversal is achieved by multiplying the gradient with a negative identity matrix, as shown in Equation 30.


(30) $$\begin{array}{l} \frac{\text{d}R_{\lambda }}{\text{d}x}=-\lambda I \end{array}$$

## 3 Experiments

### 3.1 Datasets and settings

The study utilizes the SEED dataset (Zheng and Lu, 2015) and the SEED-IV dataset (Zheng et al., 2018) for research purposes. Both of these datasets are publicly available datasets used for EEG-based emotion recognition. The SEED dataset includes 15 Chinese movie clips as stimuli for the experiments. These movie clips contain three types of emotions: positive, neutral, and negative. Each clip has a duration of ~4 min. There are a total of 15 trials in each experiment. In a session, there is a 5-s cue before each clip, followed by a self-assessment period of 45 s, and then a 15-s rest after each clip. Two movie clips with the same emotion are not presented consecutively. The EEG signals are collected using a 62-channel ESI Neuroscan system. The SEED-IV dataset comprises 72 movie clips as experimental stimuli. These movie clips include four types of emotions: happy, sad, fear, and neutral. A total of 15 participants took part in the experiment. For each participant, three experiments are conducted on different days, each containing 24 trials. In each trial, the participant watched one of the movie clips, while their EEG signals were recorded using a 62-channel ESI Neuroscan system. The EEG signals from 62 channels are recorded using the ESI Neuroscan system at a sampling rate of 1,000 Hz, which is downsampled to 200 Hz. Band-pass filtering is applied to the EEG data to remove noise and artifacts, and features such as DE are extracted from each segment in five frequency bands (δ: 1~4Hz, θ: 4~8Hz, α: 8~14Hz, β: 14~31Hz, γ: 31~50Hz).

We train and test the DSP-EmotionNet model using a Tesla V100-SXM2-32GB GPU and implement it using the PyTorch framework. The training is conducted using an Adam optimizer, and the learning rate is set to 5e-4. The batch size is set to 64, and the dropout rate is set to 0.7. The number of classes to classify for the SEED dataset is 3, while for the SEED-IV dataset, it is 4. We adopt the leave-one-subject-out (LOSO) cross-validation strategy to partition the dataset. Specifically, we use all data from 14 subjects as the training set. The remaining 1 subject is treated as an unknown subject and used as the test set. The cross-entropy loss is used as a loss function in this paper. The summary of the hyper-parameter settings is as shown in Table 1.

**Table 1:** The settings of hyper-parameters on the SEED and SEED-IV datasets are summarized.

	**SEED**	**SEED-IV**
Optimizer	Adam	Adam
Learning rate	5e-4	5e-4
Number of classes	3	4
Batch size	64	64
Loss function	Cross-entropy	Cross-entropy
Dropout rate	0.7	0.7

### 3.2 Baseline methods

In order to evaluate the effectiveness of the proposed model, a comparative analysis is conducted with several baseline methods using the SEED and SEED IV datasets. Brief introductions to each of these methods are provided below.

- SVM (Suykens and Vandewalle, 1999): Support vector machine utilizes the least squares to perform classification.
- RF (Breiman, 2001): Random forest is an ensemble learning method that integrates numerous decision trees to improve classification accuracy.
- MLP (Rumelhart et al., 1986): A multilayer perceptron represents a fundamental type of feedforward neural network, characterized by its layered structure of neurons arranged in a sequence from input to output.
- STRNN (Zhang et al., 2019): The proposed framework, known as spatial–temporal recurrent neural network (STRNN), integrates spatial and temporal data for the effective classification of human emotions.
- 3D-CNN (Zhao et al., 2020): It introduced a 3D convolutional neural network model for emotion recognition using EEG signals, which automatically extracted spatial-temporal features to achieve high classification accuracy.
- MMResLSTM (Ma et al., 2019): It proposed a Multimodal Residual LSTM Network for emotion recognition, which leveraged shared weights between different modalities to capture temporal correlations in EEG signals, thus achieving high classification accuracy.
- CDCN (Gao et al., 2021): It proposed a channel-fused dense convolutional network for EEG-based emotion recognition. This network utilizes convolutional and dense structures to process the temporal and electrode-related features of EEG signals, enhancing the model's ability to capture time dependencies and electrode correlations.
- ACRNN (Tao et al., 2020): It proposed an attention-based convolutional recurrent neural network for EEG-based emotion recognition, which utilizes channel-wise attention to dynamically weigh channels and incorporates self-attention to improve feature extraction from EEG signals.
- STFFNN (Wang et al., 2022): It introduced the Spatial-Temporal Feature Fusion Neural Network for EEG-based emotion recognition. This network combines spatial dependency learning, temporal feature learning, and feature fusion using convolutional neural networks and bidirectional LSTM, aiming to enhance emotion recognition accuracy.
- TSception (Ding et al., 2022): It proposed a novel multi-scale convolutional neural network for EEG-based emotion recognition, which captures both the temporal dynamics and spatial asymmetry of brain activity.
- MetaEmotionNet (Ning et al., 2023): It integrates spatial-frequency-temporal features into a unified network architecture and utilizes meta-learning to achieve rapid adaptation to new tasks.

### 3.3 Experimental results and analysis

Tables 2, 3 presents the cross-subject experimental results on the SEED and SEED-IV datasets, showcasing the average accuracy (ACC) and standard deviation (STD) of both the reference approaches and the proposed DSP-EmotionNet framework for emotion recognition based on EEG signals. Across the SEED dataset, our approach surpasses alternative methodologies in the inter-subject transfer scenario, achieving an ACC of 0.825 with an STD of 0.076. Regarding the SEED-IV dataset, which involves a four-category classification task, the performance of our technique is relatively lower compared to the SEED dataset. Specifically, for the SEED-IV dataset, our technique achieves an ACC of 0.659, with an STD of 0.078.

**Table 2:** Performance comparison between the baseline methods and the proposed DSP-EmotionNet on the SEED datasets.

**Method**	**ACC/STD**	**F1-score/STD**	**Kappa/STD**
RF (Breiman, 2001)	0.533/0.087	0.487/0.115	0.299/0.129
SVM (Suykens and Vandewalle, 1999)	0.531/0.109	0.468/0.137	0.296/0.163
MLP (Rumelhart et al., 1986)	0.701/0.094	0.674/0.123	0.550/0.142
STRNN (Zhang et al., 2019)	0.732/0.111	0.723/0.116	0.598/0.166
3D-CNN (Zhao et al., 2020)	0.742/0.078	0.738/0.079	0.613/0.116
MMResLSTM (Ma et al., 2019)	0.744/0.104	0.713/0.106	0.616/0.157
CDCN (Gao et al., 2021)	0.693/0.094	0.668/0.116	0.540/0.142
ACRNN (Tao et al., 2020)	0.763/0.081	0.739/0.093	0.644/0.121
STFFNN (Wang et al., 2022)	0.720/0.090	0.0710/0.095	0.579/0.136
TSception (Ding et al., 2022)	0.643/0.098	0.636/0.107	0.465/0.148
MetaEmotionNet (Ning et al., 2023)	0.775/0.088	0.772/0.087	0.663/0.132
**DSP-EmotionNet**	**0.825/0.076**	**0.824/0.072**	**0.739/0.126**

**Table 3:** Performance comparison between the baseline methods and the proposed DSP-EmotionNet on the SEED-IV datasets.

**Method**	**ACC/STD**	**F1-score/STD**	**Kappa/STD**
RF (Breiman, 2001)	0.347/0.066	0.297/0.083	0.134/0.083
SVM (Suykens and Vandewalle, 1999)	0.411/0.074	0.348/0.075	0.209/0.094
MLP (Rumelhart et al., 1986)	0.507/0.063	0.397/0.083	0.328/0.089
STRNN (Zhang et al., 2019)	0.532/0.074	0.517/0.076	0.372/0.100
3D-CNN (Zhao et al., 2020)	0.541/0.109	0.507/0.133	0.384/0.149
MMResLSTM (Ma et al., 2019)	0.511/0.114	0.461/0.112	0.348/0.149
CDCN (Gao et al., 2021)	0.545/0.131	0.521/0.150	0.393/0.172
ACRNN (Tao et al., 2020)	0.492/0.092	0.462/0.082	0.296/0.110
STFFNN (Wang et al., 2022)	0.567/0.073	0.550/0.075	0.418/0.099
TSception (Ding et al., 2022)	0.562/0.095	0.558/0.096	0.414/0.128
MetaEmotionNet (Ning et al., 2023)	0.612/0.083	0.589/0.102	0.479/0.114
**DSP-EmotionNet**	**0.659/0.078**	**0.652/0.119**	**0.542/0.108**

**Figure 4:**
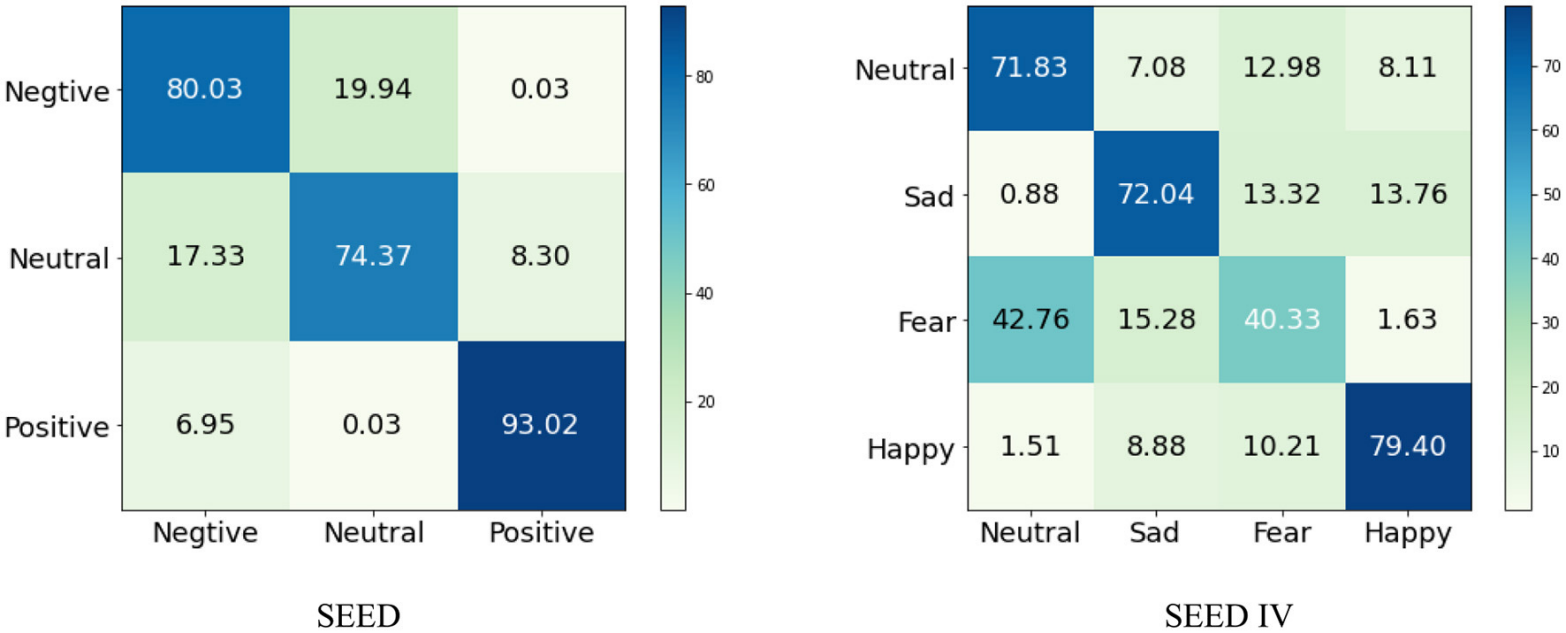
The confusion matrices of DSP-EmotionNet on SEED and SEED IV datasets.

Our proposed DSP-EmotionNet model excels in emotion recognition tasks. In contrast, traditional machine learning methods such as SVM and RF perform relatively poorly, primarily due to their inability to capture the rich information present in EEG signals. Deep learning-based CNN and RNN can better extract deep temporal or spatial features, hence methods like STRNN, 3D-CNN, CDCN, and MMResLSTM based on deep learning outperform traditional machine learning methods in terms of performance. Recently proposed methods like ACRNN, STFFNN, and TSception model both the temporal and spatial dimensions of EEG signals, improving classification stability by introducing attention mechanisms, integrating discriminative features, and capturing temporal dynamics, resulting in better results on the SEED or SEED-IV datasets. Our proposed DSP-EmotionNet model not only captures spatial local features of EEG signals but also captures the correlations between different regions of EEG signals. MetaEmotionNet utilizes two streams of spatial-temporal and spatial-frequency information, along with attention mechanisms, to comprehensively extract spatial-frequency-temporal features of EEG signals, thus achieving optimal performance in metrics. Moreover, meta-learning methods effectively enhance the adaptability of the model. Compared to the MetaEmotionNet method, our proposed DSP-EmotionNet model employs Domain Adversarial Neural Networks (DANN) to improve the generalization ability of the model. DANN technology enables the model to gradually adapt to the data distribution of new domains during the training process, thereby enhancing its generalization ability on new domains and improving the recognition rate of the model in cross-subject EEG emotion recognition tasks. Our proposed DSP-EmotionNet model not only exhibits high performance in emotion recognition tasks but also demonstrates stronger adaptability and generalization capabilities. For more detailed classification results, the confusion matrices of the proposed DSP-EmotionNet are respectively shown in Figure 4.

### 3.4 Ablation experiments

To validate the impact of different components in our proposed model on EEG emotion recognition tasks, we conduct ablation experiments on the SEED and SEED IV datasets. Our proposed method, named DSP-EmotionNet, consists primarily of three parts: spatial activity feature extractor, spatial topological feature extractor, and domain adversarial neural network. To verify the effectiveness of these three key components in our approach, we conduct ablation experiments on DSP-EmotionNet. Table 4 illustrate the impact of these three key components of DSP-EmotionNet on cross-subject EEG emotion recognition tasks. “SAE” denotes using only the spatial activity feature extractor for cross-subject EEG emotion recognition tasks. “STE” denotes using only the spatial topological feature extractor for cross-subject EEG emotion recognition tasks. “SAE-STE” represents combining the spatial activity feature extractor and spatial topological feature extractor for cross-subject EEG emotion recognition tasks, excluding domain adversarial neural network. “SAE-DANN” represents combining the spatial activity feature extractor with domain adversarial neural network for cross-subject EEG emotion recognition tasks, excluding the spatial topological feature extractor. “STE-DANN” represents combining the spatial topological feature extractor with domain adversarial neural network for cross-subject EEG emotion recognition tasks, excluding the spatial activity feature extractor.

**Table 4:** Ablation experiments on the major components of DSP-EmotionNet were conducted on the SEED and SEED IV datasets.

**Model**	**Accuracy (%)**	**Another Metric (%)**
SAE	69.7%	51.4%
STE	72.6%	55.6%
SAE-STE	77.1%	60.1%
SAE-DANN	72.2%	56.1%
STE-DANN	76.7%	59.7%
DSP-EmotionNet	82.5%	65.9%

The accuracy of “SAE” on the SEED dataset is 69.7%, and on the SEED IV dataset, it is 51.4%. In comparison, “STE” achieves an accuracy of 72.6% on the SEED dataset and 55.6% on the SEED IV dataset. This indicates that the spatial topological feature extractor performs better than the spatial activity feature extractor in cross-subject EEG emotion recognition tasks. Furthermore, “SAE-STE” outperforms “SAE” and “STE” on both the SEED and SEED IV datasets, demonstrating the effectiveness of combining EEG spatial activity features and EEG spatial topological features. On the SEED dataset, “SAE-DANN” and “STE-DANN” achieve accuracies of 72.2% and 76.7%, respectively, outperforming “SAE” and “STE”. On the SEED IV dataset, “SAE-DANN” and “STE-DANN” achieve accuracies of 56.1% and 59.7%, respectively, also outperforming “SAE” and “STE”. This indicates that DANN technology enables the model to gradually adapt to the data distribution of new domains during training, thereby improving the generalization ability of the model on new domains. DSP-EmotionNet achieves accuracies of 82.5% and 65.9% on the SEED and SEED IV datasets, respectively, surpassing the results of other methods in the ablation experiments. These results collectively demonstrate that the integration of feature fusion and domain adaptation contributes to the enhancement of model recognition performance in cross-subject EEG emotion recognition tasks.

**Figure 5:**
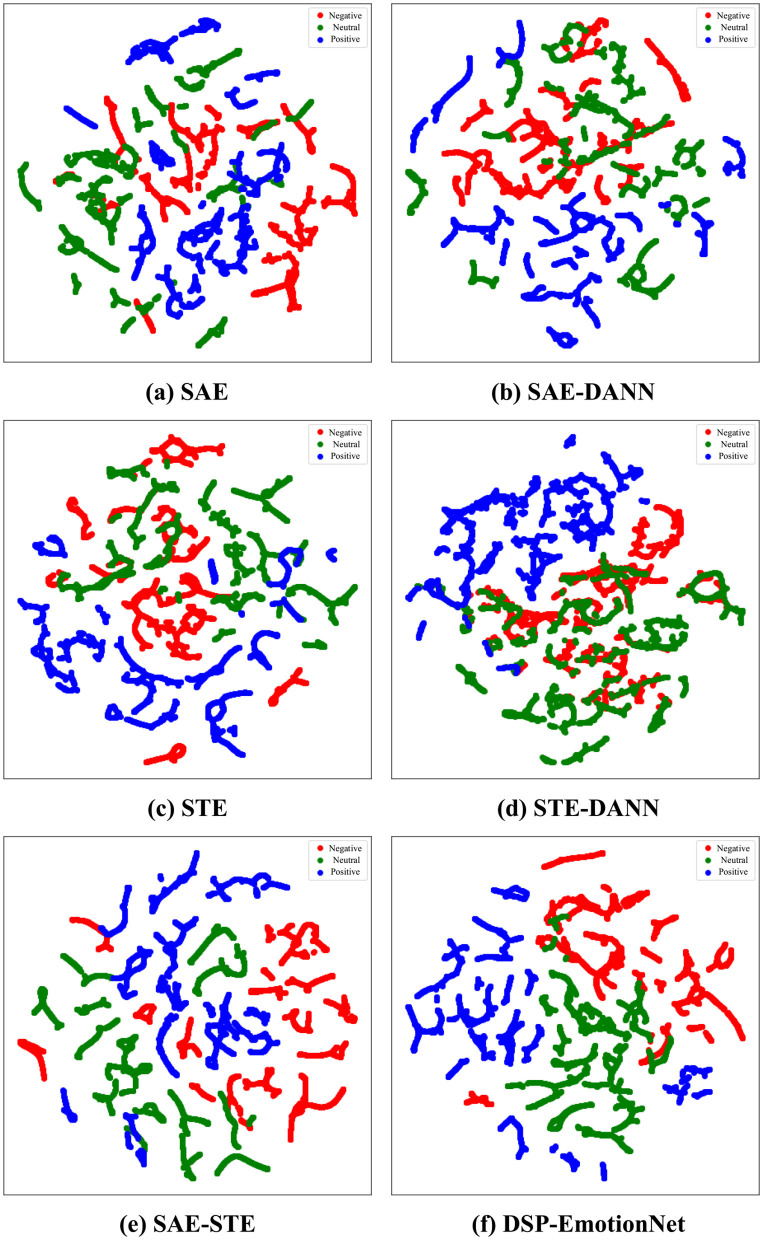
The performance of various methods in cross-subject EEG emotion recognition tasks was visualized using t-SNE. **(a)** SAE. **(b)** SAE-DANN. **(c)** STE. **(d)** STE-DANN. **(e)** SAE-STE. **(f)** DSP-EmotionNet.

To visually understand the effectiveness of DSP-EmotionNet, we randomly select a participant from the SEED dataset and use their EEG samples as the test set. We visualize the data using t-SNE (Van der Maaten and Hinton, 2008) scatter plots, as shown in Figure 5. Specifically, we select six methods for visualization experiments: “SAE”, “STE”, “SAE-STE”, “SAE-DANN”, “STE-DANN”, and “DSP-EmotionNet.” Data points are color-coded to represent three different emotions: negative emotions in red, neutral emotions in green, and positive emotions in blue. It is worth noting that the range of the data after dimensionality reduction varies depending on the participant. Here, we only show the visualization results of our method. The figure displays scatter plots for the six different methods. As shown in Figure 5a, data points corresponding to the three emotions clearly intermingle, exhibiting significant overlap. This suggests that “SAE” may face challenges in distinguishing emotions in cross-subject EEG emotion recognition tasks. In Figure 5b, the clusters appear somewhat separated, but there is still considerable overlap between emotions, especially between negative and neutral states. This indicates that although “SAE-DANN” improves upon “SAE”, it may not be sufficient for optimal emotion recognition on its own. In Figure 5c, clusters for each emotion seem more distinct compared to “SAE”, indicating that “STE” significantly enhances the discernibility of emotions. As shown in Figure 5d, clusters for positive and neutral emotions are notably different and well-separated. In Figure 5e, clusters for each emotion perform better than individual “SAE” and “STE” methods, indicating that “SAE-STE” can extract more effective features. As shown in Figure 5f, DSP-EmotionNet exhibits notably distinct and well-separated clusters for each emotion compared to “SAE-STE,” particularly the positive (blue) cluster, which is almost completely isolated from the other two emotions. This further emphasizes that the integration of feature fusion and domain adaptation significantly contributes to enhancing the recognition performance of the model in cross-subject EEG emotion recognition tasks.

## 4 Conclusion

In this paper, we introduce a domain adaptation EEG signal spatial feature perception network, named DSP-EmotionNet, for cross-subject EEG emotion recognition tasks. Initially, we extract DE features and spatially map them based on electrode position distribution to generate representations of EEG signal spatial activity features, and similarly for spatial graph mapping to produce representations of EEG signal spatial topological features. These two features serve as the input for our proposed model. Then, we design a dual-branch network, named SATFEM, utilizing a spatial activity feature extractor branch to capture EEG signal spatial activity features and a spatial topological feature extractor branch to capture EEG signal spatial topological features. The features extracted from both branches are effectively fused and classified in the feature fusion and classification layer. Finally, we employ SATFEM as the feature extractor and design a domain adaptation network to better adapt the model to the features of the target domain, thereby enhancing the accuracy of the model on cross-subject EEG emotion recognition tasks. The proposed DSP-EmotionNet achieved average recognition accuracies of 82.5% and 65.9% on the SEED and SEED-IV datasets, respectively, surpassing state-of-the-art methods. To evaluate the impact of different components in DSP-EmotionNet on the EEG emotion recognition task, we conduct ablation experiments on the SEED and SEED-IV datasets. The experimental results show that the combination of the spatial activity feature extractor branch and the spatial topological feature extractor branch can effectively enhance the capability of the model for feature extraction, and applying a domain adaptation network allows the model to better adapt to the features of the target domain, improving the generalizability of the model. The proposed DSP-EmotionNet represents a new approach to cross-subject EEG emotion recognition. This method can also be easily applied to other EEG classification tasks, such as motor imagery and sleep stage classification. However, the current model still has some limitations in practical applications. For instance, the proposed dual-branch structure has higher computational complexity compared to single-branch models, and it also lacks the capability for real-time online processing. In future work, we will investigate model compression and acceleration, as well as the real-time online capabilities of DSP-EmotionNet in cross-subject EEG emotion recognition, aiming to further enhance the generalizability and practicality of the model.

## Data Availability

The original contributions presented in the study are included in the article/supplementary material, further inquiries can be directed to the corresponding authors.

## References

[B1] Asadzadeh S. Rezaii T. Y. Beheshti S. Meshgini S. (2023). Accurate emotion recognition utilizing extracted EEG sources as graph neural network nodes. Cogn. Comput. 15, 176–189. 10.1007/s12559-022-10077-535917638

[B2] Atkinson J. Campos D. (2016). Improving bci-based emotion recognition by combining EEG feature selection and Kernel classifiers. Expert Syst. Appl. 47, 35–41. 10.1016/j.eswa.2015.10.049

[B3] Bahari F. Janghorbani A. (2013). “EEG-based emotion recognition using recurrence plot analysis and K nearest neighbor classifier,” in 2013 20th Iranian Conference on Biomedical Engineering (ICBME) (Tehran: IEEE), 228–233.

[B4] Breiman L. (2001). Random forests. Machine Learn. 45, 5–32. 10.1023/A:1010933404324

[B5] Chakravarthi B. Ng S.-C. Ezilarasan M. Leung M.-F. (2022). Eeg-based emotion recognition using hybrid CNN and LSTM classification. Front. Comput. Neurosci. 16:1019776. 10.3389/fncom.2022.1019776 36277613 PMC9585893

[B6] Cimtay Y. Ekmekcioglu E. Caglar-Ozhan S. (2020). Cross-subject multimodal emotion recognition based on hybrid fusion. IEEE Access 8, 168865–168878. 10.1109/ACCESS.2020.3023871

[B7] Ding Y. Robinson N. Zhang S. Zeng Q. Guan C. (2022). Tsception: capturing temporal dynamics and spatial asymmetry from EEG for emotion recognition. IEEE Trans. Affect. Comput. 2022:3169001. 10.1109/TAFFC.2022.3169001

[B8] Doma V. Pirouz M. (2020). A comparative analysis of machine learning methods for emotion recognition using EEG and peripheral physiological signals. J. Big Data 7, 1–21. 10.1186/s40537-020-00289-7

[B9] Ganin Y. Lempitsky V. (2015). “Unsupervised domain adaptation by backpropagation,” in Proceedings of the 32nd International Conference on Machine Learning, eds. F. Bach and D. Blei (Lille: PMLR), 1180–1189.

[B10] Gao Z. Wang X. Yang Y. Li Y. Ma K. Chen G. (2021). A channel-fused dense convolutional network for EEG-based emotion recognition. IEEE Trans. Cogn. Dev. Syst. 2020, 945–954. 10.1109/TCDS.2020.297611232260445

[B11] Jia Z. Lin Y. Cai X. Chen H. Gou H. Wang J. (2020). “Sst-emotionnet: Spatial-spectral-temporal based attention 3d dense network for eeg emotion recognition,” in Proceedings of the 28th ACM International Conference on Multimedia, 2909–2917.

[B12] Jia Z. Lin Y. Wang J. Feng Z. Xie X. Chen C. (2021). “HetEmotionNet: two-stream heterogeneous graph recurrent neural network for multi-modal emotion recognition,” in Proceedings of the 29th ACM International Conference on Multimedia, 1047–1056.

[B13] Jin Y.-M. Luo Y.-D. Zheng W.-L. Lu B.-L. (2017). “EEG-based emotion recognition using domain adaptation network,” in 2017 International Conference on Orange Technologies (ICOT) (Singapore: IEEE), 222–225.

[B14] Kwon Y.-H. Shin S.-B. Kim S.-D. (2018). Electroencephalography based fusion two-dimensional (2D)-convolution neural networks (CNN) model for emotion recognition system. Sensors 18:1383. 10.3390/s18051383 29710869 PMC5982398

[B15] Li J. Qiu S. Du C. Wang Y. He H. (2019). Domain adaptation for EEG emotion recognition based on latent representation similarity. IEEE Trans. Cogn. Dev. Syst. 12, 344–353. 10.1109/TCDS.2019.2949306

[B16] Li J. Zhang Z. He H. (2018). Hierarchical convolutional neural networks for EEG-based emotion recognition. Cogn. Comput. 10, 368–380. 10.1007/s12559-017-9533-x

[B17] Li Y. Wang L. Zheng W. Zong Y. Qi L. Cui Z.. (2020). A novel bi-hemispheric discrepancy model for EEG emotion recognition. IEEE Trans. Cogn. Dev. Syst. 13, 354–367. 10.1109/TCDS.2020.2999337

[B18] Ma J. Tang H. Zheng W.-L. Lu B.-L. (2019). “Emotion recognition using multimodal residual LSTM network,” in Proceedings of the 27th ACM International Conference on Multimedia, 176–183.

[B19] Ning X. Wang J. Lin Y. Cai X. Chen H. Gou H.. (2023). MetaemotionNet: spatial-spectral-temporal based attention 3D dense network with meta-learning for EEG emotion recognition. IEEE Trans. Instrument. Measur. 2023:3338676. 10.1109/TIM.2023.3338676

[B20] Ramzan M. Dawn S. (2023). Fused CNN-LSTM deep learning emotion recognition model using electroencephalography signals. Int. J. Neurosci. 133, 587–597. 10.1080/00207454.2021.1941947 34121598

[B21] Rumelhart D. E. Hinton G. E. Williams R. J. (1986). Learning internal representations by error propagation, parallel distributed processing, explorations in the microstructure of cognition. Biometrika 71, 599–607.

[B22] Suykens J. A. K. Vandewalle J. (1999). Least squares support vector machine classifiers. Neural Proc. Lett. 9, 293–300. 10.1023/a:1018628609742 11972910

[B23] Tan C. Ceballos G. Kasabov N. Puthanmadam Subramaniyam N. (2020). Fusionsense: emotion classification using feature fusion of multimodal data and deep learning in a brain-inspired spiking neural network. Sensors 20:5328. 10.3390/s20185328 32957655 PMC7571195

[B24] Tao W. Li C. Song R. Cheng J. Liu Y. Wan F.. (2020). EEG-based emotion recognition via channel-wise attention and self attention. IEEE Trans. Affect. Comput. 2020:3025777. 10.1109/TAFFC.2020.3025777

[B25] Van der Maaten L. Hinton G. (2008). Visualizing data using T-SNE. J. Machine Learn. Res. 9, 2579–2605.

[B26] Velickovic P. Cucurull G. Casanova A. Romero A. Lio P. Bengio Y.. (2017). Graph attention networks. Stat 2017:10903. 10.48550/arXiv.1710.10903

[B27] Wang F. Wu S. Zhang W. Xu Z. Zhang Y. Wu C.. (2020). Emotion recognition with convolutional neural network and EEG-based efdms. Neuropsychologia 146:107506. 10.1016/j.neuropsychologia.2020.107506 32497532

[B28] Wang X.-W. Nie D. Lu B.-L. (2011). “EEG-based emotion recognition using frequency domain features and support vector machines,” in Neural Information Processing: 18th International Conference, ICONIP 2011, Shanghai, China, November 13-17, 2011, Proceedings, Part I 18 (Berlin: Springer), 734–743.

[B29] Wang Y. Liu J. Ruan Q. Wang S. Wang C. (2021). Cross-subject EEG emotion classification based on few-label adversarial domain adaption. Exp. Syst. Appl. 185:115581. 10.1016/j.eswa.2021.115581

[B30] Wang Z. Wang Y. Zhang J. Hu C. Yin Z. Song Y. (2022). Spatial-temporal feature fusion neural network for EEG-based emotion recognition. IEEE Trans. Instr. Measur. 71, 1–12. 10.1109/TIM.2022.3165280

[B31] Xing X. Li Z. Xu T. Shu L. Hu B. Xu X. (2019). SAE+ LSTM: a new framework for emotion recognition from multi-channel EEG. Front. Neurorobot. 13:37. 10.3389/fnbot.2019.00037 31244638 PMC6581731

[B32] Zhang T. Zheng W. Cui Z. Zong Y. Li Y. (2019). Spatial—temporal recurrent neural network for emotion recognition. IEEE Trans. Cybernet. 839–847. 10.1109/TCYB.2017.2788081 29994572

[B33] Zhang X. Huang D. Li H. Zhang Y. Xia Y. Liu J. (2023). Self-training maximum classifier discrepancy for EEG emotion recognition. CAAI Trans. Intell. Technol. 2023:12174. 10.1049/cit2.12174

[B34] Zhao Y. Yang J. Lin J. Yu D. Cao X. (2020). “A 3D convolutional neural network for emotion recognition based on EEG signals,” in 2020 International Joint Conference on Neural Networks (IJCNN) (Glasgow: IEEE), 1–6.

[B35] Zheng W.-L. Liu W. Lu Y. Lu B.-L. Cichocki A. (2018). Emotionmeter: a multimodal framework for recognizing human emotions. IEEE Trans. Cybernet. 49, 1110–1122. 10.1109/TCYB.2018.2797176 29994384

[B36] Zheng W.-L. Lu B.-L. (2015). Investigating critical frequency bands and channels for EEG-based emotion recognition with deep neural networks. IEEE Trans. Auton. Ment. Dev. 7, 162–175. 10.1109/TAMD.2015.2431497

[B37] Zhou X. Liu C. Zhai L. Jia Z. Guan C. Liu Y. (2023). Interpretable and robust AI in EEG systems: a survey. arXiv preprint arXiv:2304.10755. 10.48550/arXiv.2304.10755

